# A Single Dose of Marine *Chlorella vulgaris* Increases Plasma Concentrations of Lutein, β-Carotene and Zeaxanthin in Healthy Male Volunteers

**DOI:** 10.3390/antiox10081164

**Published:** 2021-07-22

**Authors:** Ana Teresa Serra, Sandra D. Silva, Luís Pleno de Gouveia, Agostinho M. R. C. Alexandre, Carolina V. Pereira, Ana Barbara Pereira, Ana Carvalho Partidário, Nuno Elvas Silva, Torsten Bohn, Vanessa S. S. Gonçalves, Gonçalo Real, Pedro Escudero, Naiara Fernández, Ana A. Matias, Maria Rosário Bronze

**Affiliations:** 1 iBET, Instituto de Biologia Experimental e Tecnológica, Apartado 12, 2780-901 Oeiras, Portugal; ssilva@ibet.pt (S.D.S.); agostinho@ibet.pt (A.M.R.C.A.); carolina.pereira@ibet.pt (C.V.P.); anab.pereira@ibet.pt (A.B.P.); naiara.fernandez@ibet.pt (N.F.); amatias@ibet.pt (A.A.M.); 2 Instituto de Tecnologia Química e Biológica António Xavier, Universidade Nova de Lisboa (ITQB NOVA), 2780-157 Oeiras, Portugal; 3 iMed, Faculdade de Farmácia da Universidade de Lisboa, Av das Forças Armadas, 1649-019 Lisboa, Portugal; lgouveia@campus.ul.pt (L.P.d.G.); nmens@ff.ulisboa.pt (N.E.S.); 4 INIAV, Instituto Nacional de Investigação Agrária e Veterinária, I.P., Avenida da República, Quinta do Marquês, 2780-157 Oeiras, Portugal; ana.partidario@iniav.pt; 5 Nutrition and Health Research Group, Department of Population Health, Luxembourg Institute of Health, 1 A–B, rue Thomas Edison, L-1445 Strassen, Luxembourg; torsten.bohn@lih.lu; 6 Buggypower (Portugal), Lda., Alameda dos Oceanos, Parque das Nações, 1990-203 Lisboa, Portugal; vgoncalves@buggypower.eu (V.S.S.G.); greal@buggypower.eu (G.R.); pescudero@buggypower.eu (P.E.)

**Keywords:** green algae, xanthophylls, polyunsaturated fatty acids (PUFA), kinetic study, human intervention, plant bioactives, food supplement

## Abstract

The beneficial health effects of *Chlorella vulgaris* have been associated with the presence of several nutrients and antioxidants, including carotenoids. However, the in vivo bioavailability of *Chlorella* is still poorly evaluated. In this work, a human intervention study was conducted in 11 healthy men to evaluate the bioavailability of carotenoids within 3 days after the intake of a single dose (6 g) of dried marine *Chlorella vulgaris* containing lutein (7.08 mg), β-carotene (1.88 mg) and zeaxanthin (1.47 mg). Subjects were instructed to follow a low carotenoid diet during the experimental phase, starting 1 week earlier. On the day of the experiment, dried microalgae formulated in vegetarian hard capsules were ingested, and blood samples were collected up to 72 h for the analysis of plasma carotenoids concentration by high-performance liquid chromatography with diode-array detection. For all carotenoids, the estimated AUC and C_max_ values were significantly different from zero (*p* < 0.05), indicating that a single dose of marine *Chlorella vulgaris* increased plasma concentrations of lutein (C_min_-corrected AUC = 1002 µg·h/L, C_max_ = 20.4 µg/L), β-carotene (AUC = 1302 µg·h/L, C_max_ = 34.9 µg/L) and zeaxanthin (AUC = 122.2 µg·h/L, C_max_ = 3.4 µg/L). The bioavailability of other compounds, namely, polyunsaturated fatty acids and trace elements, was also assessed post-prandial for the first time, showing that linoleic acid, docosahexaenoic acid and iodine were absorbed after microalgae intake. These findings support the use of *Chlorella vulgaris* as a source of carotenoids, PUFA and essential trace elements with associated health benefits.

## 1. Introduction

*Chlorella* is an unicellular green microalga naturally found in marine and freshwater environments. It is capable of photosynthesis using solar energy, carbon dioxide as a carbon source and water. Being regarded first as a feedstock for biofuel production, their outstanding nutritional characteristics [1] make them an optimal supplement to promote human health. *Chlorella* species contain several macro- and micro-nutrients, such as carbohydrates, proteins, essential amino acids, polyunsaturated fatty acids (PUFA), vitamins (B12 and B9), minerals, dietary fiber and carotenoids [2]. Due to their nutritional and phytochemical composition, *Chlorella* species have been reported to potentially prevent lifestyle-related diseases, including cardiovascular diseases [3,4], neurological disorders [5] and to improve the immune system [6] and antioxidant status [7] in humans. In a recent meta-analysis, *Chlorella* supplementation was found to improve total and low-density lipoprotein cholesterol levels, systolic and diastolic blood pressure and fasting blood glucose in humans [8].

The beneficial health effects of *Chlorella* have been associated with the presence and synergism between multiple nutrient and antioxidant compounds, including carotenoids [9]. Ryu et al. showed that *Chlorella vulgaris* (in a dose of 5 g/day) appears to have beneficial health effects on the serum lipid profiles of mildly hypercholesterolemic subjects due in part to the improvement of serum carotenoid profiles, suggesting that these microalgae should be included in the list for recommending heart-healthy dietary supplements [4]. Taiki and co-authors showed that carotenoids from *Chlorella vulgaris* could be transferred from plasma lipoprotein particles to the erythrocyte membrane, which suggests that *Chlorella* intake would be effective for improving carotenoids’ concentrations in human erythrocytes, acting as important antioxidants for these cells [10]. The main carotenoids identified in *Chlorella* species include lutein (the most abundant), β-carotene and zeaxanthin [4]. However, their bioavailability, which is dependent on the type of food matrix [11], is still poorly evaluated. *Chlorella vulgaris* is also a good source of dietary fiber [9]; this may have a hampering effect on carotenoid absorption [12].

In this investigation, a human post-prandial intervention study was conducted in healthy male volunteers to evaluate the bioavailability of carotenoids within 3 days after the intake of a single dose (6 g) of dried marine *Chlorella vulgaris*. The bioavailability of other compounds, namely, PUFA (linoleic acid, α-linolenic acid, eicosapentaenoic acid (EPA) and docosahexaenoic acid (DHA)) and trace elements (Se and I) was also assessed for the first time from this matrix.

## 2. Materials and Methods

### 2.1. Chemicals and Standards

Lutein (0306 S, ≥95%), zeaxanthin (0307 S, ≥98%) and β-apo-8′-carotenal (0330) were purchased from Extrasynthese (France) and β-carotene (72355-40-7, 97%) was from Sigma (St. Louis, MO, USA). Chemicals and reagents used were of analytical grade and included heptadecanoic acid (Sigma Aldrich, USA), chloroform (Carlo Erba. Val de Reuil, France), NaOH 98% (Acros Organics, Göteborg, Sweden), methanol (Fisher Chemical, Merelbeke, Belgium), BF_3_ 14% in methanol (Sigma Aldrich, USA), isooctane 99+% (Sigma Aldrich, Darmstadt, Germany), BHT (The British Drug house Ltd. Poole, London, England), ethanol (Fisher Chemical, Merelbeke, Belgium), butanol (Sigma-Aldrich, St. Louis, MO, USA), ethyl acetate (Carlo Erba, Spain), acetonitrile (Fisher Chemical, USA) and triethylamine (Sigma-Aldrich, St. Louis, MO, USA).

### 2.2. Participants

Eleven healthy subjects were included in this study. To exclude any potential estrogen-related impact on carotenoid absorption, only male subjects were recruited [13,14,15]. A complete list of the inclusion and exclusion criteria is described in Supplementary Materials. Volunteers recruited for the study had a mean age of 30.5 ± 4.2 years old (ranging from 26 to 38 years old) and a body mass index (BMI) of 24.6 ± 2.6 kg/m^2^ (ranging from 20.6 to 27.8 kg/m^2^). The volunteers were not consumers of carotenoid, vitamin and/or PUFA supplements and were not regular consumers of microalgae products. Ten volunteers were lean non-smokers and one volunteer consumed 7 cigarettes/day (exclusion criteria was more than 10 cigarettes per day).

### 2.3. Test Product

The test product was freeze-dried biomass of marine *Chlorella vulgaris* strain UTEX 580 (belonging to the culture collection of algae at the University of Texas at Austin). Marine microalgae were produced by Buggypower (Portugal), Lda, using a patent-protected technology. Briefly, *Chlorella vulgaris* was grown in filtered seawater in controlled outdoor closed photobioreactors (proprietary process) under photoautotrophic conditions. The marine microalga *Chlorella vulgaris* was encapsulated in vegetarian hard capsules (YourSupplements, Stockport, UK), made from pullulan, a natural water-soluble polysaccharide in a sterile atmosphere. Each capsule contained 600 mg of dried microalgae. A dose of 6 g (10 capsules) corresponded to the following composition in carotenoids, PUFA and mineral salts: lutein-7.08 mg, β-carotene-1.88 mg, zeaxanthin-1.47 mg; linoleic acid-64.8 mg, α-linolenic acid-87.0 mg, EPA-3.30 mg, DHA-2.88 mg, Se-0.3 µg, I-79.2 µg. The nutritional content of the test product is presented in Table S1 (Supplementary Materials).

### 2.4. Protocol

**Figure 1:**
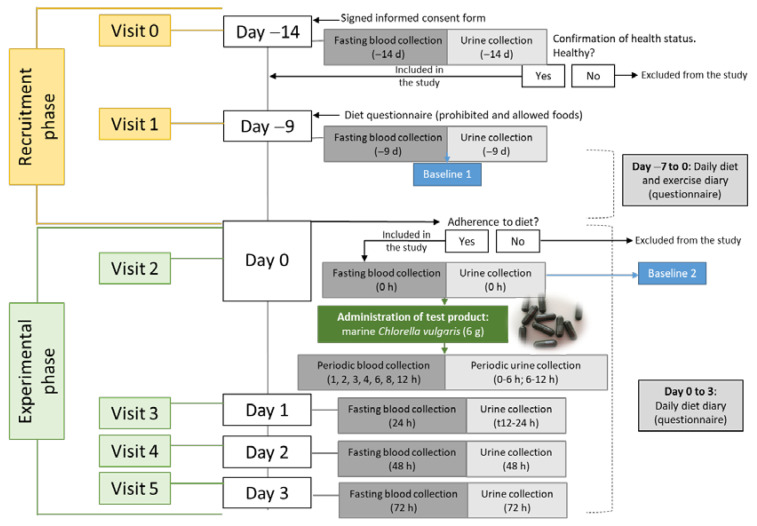
Diagram of the study.

This study is a phase I clinical study (bioavailability and safety), non-controlled and single dose. All subjects gave their written informed consent for inclusion before their participation in the study. The study was conducted in accordance with the Declaration of Helsinki, and it was approved by the local Ethics Committee of the clinic (Cintramédica II—Serviços de Saúde Lda) with the reference F6H-CT1/2018-BioavailabilityCHSP and EudraCT number 2018-001195-38. The diagram of the study is presented in Figure 1.

At day −14 (visit 0), subjects underwent a clinical checkup, and a fasting blood sample was collected to measure the baseline values of carotenoids (lutein, β-carotene and zeaxanthin) and fatty acid concentrations. At day −9 (visit 1), volunteers arrived at the clinic in the morning, and an initial fasting blood sample was collected to measure the concentration of carotenoids, fatty acids and trace elements (Se). A urine sample was also collected to evaluate the concentrations of I and Se. Volunteers were asked to maintain their usual lifestyle (exercise, sleep, daily work) during the following week and to avoid the consumption of specific foods enriched in carotenoids and fatty acids (a list of prohibited and allowed foods were provided, Table S2) during the last 7 and 3 days, respectively, before the beginning of the study and during the 3 days of the experimental phase. Volunteers were also asked to register their daily food intake and type and duration of physical exercise. On day 0 (visit 2), volunteers arrived at the clinic in the morning (08:00–10:00) after a 12 h overnight fasting and a fasting blood sample and urine were collected. After, each subject took a single *Chlorella vulgaris* dose (6 g) (total fat = 0.53 mg) within 10 capsules with a meal: wheat and rye bread (40 g) and red fruit jam (10 g) and water (energy: 115.2 kcal; total fat: 1.43 g; total carbohydrates: 23.0 g; protein: 3.7 g). Blood samples were taken at the following time points after microalga intake: 1, 2, 3, 4, 6, 8 and 12 h. Meals (low in carotenoids and fatty acids) composed of roasted chicken and rice and one apple (energy: 462 kcal; total fat: 14.7 g; total carbohydrates: 55.2 g; protein: 30 g) and bread with ham and cheese and yogurt (energy: 375.4 kcal; total fat: 18.8; total carbohydrates: 27.8 g; protein: 22.6 g) were offered to all participants at 4 and 6 h, respectively, after microalga intake. Complete urine samples were collected from all volunteers for 24 h post-dose in three intervals: 0–6 h, 6–12 h and 12–24 h. On days 1, 2 and 3 (visits 3–5) after microalga intake, fasting blood and urine samples were collected. All blood samples were drawn from a forearm vein and processed as described below. Urine samples collected at visits 1, 2, 3, 4 and 5 were refrigerated until analysis.

### 2.5. Analysis of Biological Samples

#### 2.5.1. Carotenoids Analysis in Blood Samples

For plasma separation, blood samples were collected in tubes with EDTA K2 and centrifuged (500× *g*). Plasma samples were aliquoted and stored at −80 °C until analysis. The quantification of plasma carotenoids was performed by high-performance liquid chromatography with diode-array detection (HPLC-DAD) using a methodology adapted from the literature [16,17,18]. Briefly, plasma (100 µL) was extracted with ethanol/n-butanol (1:1, *v*/*v*, 200 µL) containing β-apo-8′-carotenal as an internal standard. After incubation at −20 °C for 30 min, the mixture was centrifuged (21,000× *g* for 3 min) and 20 µL of the clear supernatant was analyzed on a Waters^®^ HPLC Alliance using a LiChrospher^®^ 100 RP-185 μm (250 × 4.0 mm) column at 450 nm as previously described in detail [18]. Pure carotenoid standard mixtures were prepared and analyzed using the same analytical conditions as for the sample for quantification. Results are expressed in µg·L^−1^ of plasma.

#### 2.5.2. PUFA Analysis in Blood Samples

Plasma lipids from stored frozen plasma aliquots were extracted by the Bligh and Dyer method [19]. Fatty acid methyl esters for gas chtomatography with flame ionization detector (GC-FID) analysis were prepared by a saponification step (methanolic NaOH) followed by acid esterification with BF_3_ in methanol, according to ISO 5509:2000 [20], and heptadecanoic acid was used as internal standard. A liquid–liquid extraction was carried out with isooctane containing BHT (5 µg/mL) as preservative, and the samples were stored at −20 °C, protected from light until further analysis. PUFA analysis was performed using a Thermo Scientific TRACE GC Ultra (Thermo Scientific, Milano, Italy) GC-FID. The separation of sample components was achieved using a J&W DB-23 capillary column (Agilent Technologies, Inc., Santa Clara, CA, USA), 60 m × 0.25 mm internal diameter and 0.25 μm phase thickness. GC-FID analysis conditions and fatty acids identification were described previously [21] and results are expressed as mg·L^−1^ of plasma.

#### 2.5.3. Se and I Analysis in Blood and Urine

Blood samples collected at visits 1 and 2 and urine samples collected at visits 1 to 5 were refrigerated, followed by shipment to Laboratorio de Análisis Echevarne. Se and I analysis in blood and urine was performed by inductively coupled plasma mass spectrometry (ICP-MS). Results are expressed as µg Se/L and µg of Se or I/g of creatinine for plasma and urine samples, respectively. The concentration of creatinine in urine was determined by UV spectrophotometry.

### 2.6. Statistical Analysis

A frequency distribution analysis was performed for fatty acids, carotenoids and trace elements. Data processing and statistical analysis were performed using Phoenix WinNonlin v.8.1 (Certara, Princeton, NJ, USA) and Unscrambler X 10.4 (Camo software, Trondheim, Norway). The concentration of compound vs. time points and derived parameters, namely, the trapezoidal AUC (area under the curve), the C_max_ and the t_max_ were estimated using the standard methodology as followed when assessing drug pharmacokinetics [22,23].

*t*-tests (paired samples and/or independent) were used to establish statistical significance on the observed changes in the data. Descriptive statistics are presented as means and SEM (standard error of the mean), and 90% confidence levels of the means (either geometric or arithmetic, depending on the data and assumptions made) was considered. The Se plasma concentrations at 24 h were corrected by subtracting the concentrations at *t* = 0 h. The post-dose plasma concentrations of carotenoids were corrected by subtracting the lowest observed value (C**_min_**) up to 6 h after supplement administration. These corrected values were statistically tested for a mean value different from zero by using a *t*-test. The I and Se urine concentrations at 24, 48 and 72 h were corrected by subtracting the concentration at *t* = 0 h. The AUC and C_max_ values were log-transformed as they were shown to be log-normally distributed according to the Shapiro–Wilk test (*p* > 0.05). These corrected values were statistically tested for a mean value different from zero by using a two-way ANOVA. A *p*-value below 0.05 (2-sided) was considered statistically significant.

## 3. Results and Discussion

### 3.1. Bioavailability of Carotenoids

The bioavailability of carotenoids from a single dose of *Chlorella* *vulgaris* in humans was only reported by Shibata and Hayakawa, who demonstrated that 3 g or 6 g of this microalga increased the serum concentration of lutein by up to 66% for 3 days after the intake [24]. The high serum lutein concentration observed could be related to the simultaneous administration of olive oil, which has already been described to improve the intestinal bioaccessibility, absorption kinetics and bioavailability of carotenoids in vivo [25,26]. Therefore, in our work, the diet of the volunteers during the intervention study was depleted of olive oil and other fat products to better investigate the bioavailability of carotenoids from this microalga alone, in addition to other compounds present in the matrix, especially PUFA.

Table 1 shows that the plasma concentrations of carotenoids (lutein, β-carotene and zeaxanthin) of subjects at the recruitment phase and start of the diet (days -14 and -9, respectively, before the intake of the controlled diet) are within the range of values reported in the literature by Riso and Stuetz [18,27]. Moreover, these values were lower than those described to be associated with a healthy and varied diet (about 268 µg/L of β-carotene and 188 µg/L of lutein and zeaxanthin) [28], indicating that the volunteers were adopting a poor carotenoid diet before the beginning of the study. The decrease observed between these values and the baseline (day 0) for all carotenoids confirmed that the volunteers also followed the low-carotenoid diet proposed for the week before the experimental phase. The average plasma carotenoid concentrations of volunteers at baseline (day 0) were significantly lower than at the previous assessments (average reductions for lutein, β-carotene and zeaxanthin were 11, 17 and 9%, respectively) (two-way ANOVA, lutein: *p* < 0.01, β-carotene: *p* = 0.02, zeaxanthin: *p* < 0.01).

After the marine *Chlorella vulgaris* intake, plasma concentration of carotenoids, considered as a better indicator of recent exposure [29,30], was accessed for 3 successive days. For most of the volunteers, the plasma concentration of carotenoids was higher at the baseline (*t* = 0) than on the following timepoints of the study (from 0.5 to 72 h), and the lowest concentrations were obtained between time points 2 h and 6 h after microalga intake (Figures S1–S3). These profiles can likely be related to the increased dietary fat intake during the lunch (14.7 g) and afternoon snack (18.8 g) at these time points, which promoted the absorption of carotenoids [31]. 

Additionally, the presence of a lag phase between 2 and 6 h after intake could be explained by the independent contribution of several factors: (i) plasma dilution effect derived from water and fluid intake (>200 mL) after baseline, following test meal consumption (as reported by other authors, the ingestion of water after an overnight fast decreased blood hemoglobin concentration and plasma osmolality [32,33]); (ii) necessary time for the dissolution of the pullulan hard capsules and the dispersion of the microalga in the gastric medium; (iii) slow gastric emptying promoted by the ingestion of a meal after microalga intake, which can last up to 2–3 h after a solid meal [34]; (iv) low solubility of carotenoids in water and in the gastric and enteric fluids, taking additional time for the transition from lipid droplets to mixed micelles, from which carotenoids are potentially bioavailable.

**Figure 2:**
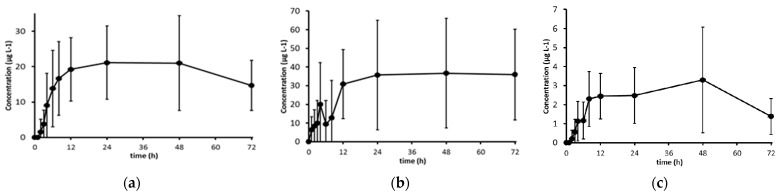
Average profile of plasma concentrations of lutein (**a**), β-carotene (**b**) and zeaxanthin (**c**) of the 11 volunteers. Error bars represent SEM. Shown values represent C_min_-subtracted concentrations.

Taking into account all these considerations, the profiles of plasma carotenoid concentration for each volunteer were recalculated, assuming that the concentration at baseline is the minimum concentration (C_min_) observed for each volunteer and carotenoid. Therefore, for each volunteer, AUC was calculated for the range (t_min_, t_72h_) by subtracting the C_min_ from the other concentration values. Figure 2 illustrates the average profiles of plasma carotenoids concentrations (C_min_ -corrected concentrations) for the 11 volunteers, and Table 2 presents the AUC, C_max_ and t_max_ calculated for each carotenoid.

Results showed that for all carotenoids, the values of AUC and C_max_ were significantly different from zero (*p* < 0.01), indicating that a single dose of marine *Chlorella vulgaris* (6 g) increased plasma concentrations of lutein, β-carotene and zeaxanthin in healthy subjects. For lutein, the major carotenoid identified in marine *Chlorella vulgaris* (Table 2), the C_max_ achieved in this study per mg of ingested lutein (2.88 µg·L^−1^) was lower than the value reported by Shibata and Hayakawa (7.23 µg·L^−1^) [24], and this difference could be explained by the absence of olive oil in the meal. When compared with other food matrices rich in lutein, the range of C_max_ obtained herein per mg of lutein intake (0.7–12.2 µg·L^−1^) are within the values reported for a dose of broccoli (200 g) (5.0 µg·L^−1^) and spinach (150 g) (10.5 µg·L^−1^) [27].

Our study reports, for the first time, the bioavailability data of the minor carotenoids after a single intake of *Chlorella vulgaris*, demonstrating higher AUC and C_max_ values for β-carotene and lower values for zeaxanthin (Table 2). These results differ from the ones reported by Jung and co-authors, where no significant increase in plasma β-carotene and zeaxanthin concentration relative to the placebo was observed over 6 weeks of daily supplementation of *Chlorella vulgaris* [30]. This could be explained by the lower content in β-carotene and zeaxanthin (up to two and four times, respectively) of the ingested microalga.

The average values of t_max_ of carotenoids ranged from 19.0 to 34.9 h according to the type of compounds, and the high inter-individual variability observed in this intervention study could be derived from host factors such as lifestyle habits and genetic variations (e.g., single nucleotide polymorphisms) of each volunteer as described by other authors [13].

### 3.2. Bioavailability of PUFA

**Figure 3:**
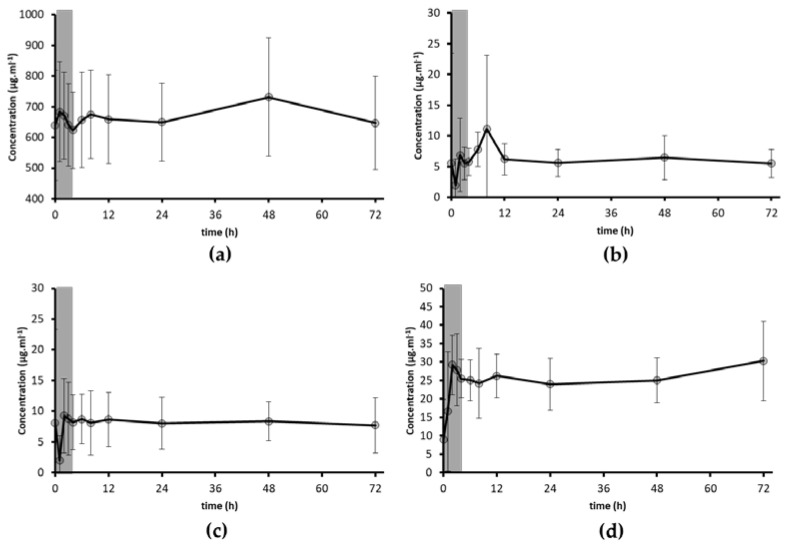
Average profile of plasma concentrations of linoleic acid (**a**), α-linolenic acid (**b**), EPA (**c**) and DHA (**d**) for the 11 volunteers. Grey area represents the first 4 h after microalgae intake. Error bars represent SEM.

Data analysis of plasma concentrations of PUFA, namely, linoleic acid, α-linolenic acid, EPA and DHA, was also performed over the 72 h of the study (Figure 3). It is important to mention that despite all the volunteers following the list of prohibited and allowed foods, the intake of target PUFA after the first meal was already higher than the dose provided by the 6 g of *Chlorella vulgaris*. Therefore, only the plasma concentration values recorded in the first 4 h were related to the microalga intake. Our results showed that the main increases were observed for the linoleic acid and DHA, indicating that a single dose of marine *Chlorella vulgaris* (6 g) may increase the plasma concentrations of these PUFA after the first 4 h of microalga intake. Up to date, there are no studies reporting the bioavailability of PUFA from microalgae, including *Chlorella* species, in humans. The majority of the bioavailability studies were performed with fish oils where the tested doses of EPA and DHA were higher (up to 500 times) [35] than the amounts reported herein for the marine microalga. In that study, the C_max_ of EPA and DHA were achieved at 2.6 h, which are within the range of values observed in our work (Figure 3).

### 3.3. Bioavailability of Trace Elements: Se and I

Plasma and urine concentrations of selenium (Se) and urine concentration of iodine (I) were also analyzed before and after 24 h of marine *Chlorella vulgaris* intake. For Se, results showed that there are no significant differences between plasma concentrations of this trace element at the different time points (day −9: 152.7 ± 15.6 µg/L; day 0: 159.5 ± 20.2; day 1: 161.4 ± 19.6 µg/L), indicating that the intake of this microalga did not increase plasma concentration of Se. Accordingly, no variation in Se excreted in urine was observed during 72 h after microalgae ingestion (Figure S4, Table S3). Although there are no studies on the bioavailability of Se from microalgae in humans, it was demonstrated that Se-deficiency in rats could be alleviated by oral supplementation with Se-rich microalgae, namely, *Arthrospira* (spirulina) [36]. For I, the intake of marine *Chlorella vulgaris* slightly increased its excretion in urine as the AUC determined between 0 and 72 h (527 ± 782 µg·h/g of creatinine, Table S3) was significantly higher than zero (*p* < 005). These data suggest that this trace element was absorbed after the intake of a single dose (6 g) of marine *Chlorella vulgaris*. It is important to note that the quantity of I administered in this study (79.2 µg) is below the Adequate Intake (AI) values recommended by European Safety Authority (EFSA) (150 µg/day for adults; 70–130 µg/day for 7–11 months infants and children; 200 µg/day for pregnant and lactating women) [37] and the tolerable upper limit established by American Thyroid Association (ATA) (1000 µg/day) [38] and by EFSA (600 µg/day) [39].

There are several limitations in this study. First, the study was carried out with a homogenous male group (average age of 30.5 ± 4.2 years old), which limits the extrapolation of the results to the general population. In addition, the inclusion of a placebo group/arm together with high control of meals (e.g., the same meals for all volunteers over the experimental phase) would have potentially strengthened the study by providing bioavailability data of PUFA over 72 h after microalga intake. Another limitation of the study is that the carotenoids were only quantified in plasma samples. Even though the plasma carotenoids concentration is considered a good indicator of recent exposure [29,30], the analysis of the triacylglycerol-rich lipoprotein fraction should also be considered in future studies to better discriminate the newly absorbed and circulating carotenoids.

## 4. Conclusions

In conclusion, this study demonstrated that a single dose of 6 g of marine *Chlorella vulgaris* significantly increased plasma concentrations of carotenoids, namely, lutein, β-carotene and zeaxanthin, for 3 days. The bioavailability of essential PUFA and essential trace elements was also assessed, showing for the first time that linoleic acid, DHA and I are absorbed after a single-dose microalga intake. The data generated herein represent a first step towards the design of further intervention studies to evaluate the health-promoting effects of marine *Chlorella vulgaris* and to develop formulation strategies to improve the bioavailability of its carotenoids, PUFA and other important micro- and macronutrients. Nevertheless, these findings support not only the use of *Chlorella vulgaris* as a carotenoid source, corroborating the outcomes of other researchers, but they also demonstrate that these microalgae can be a source of PUFA and essential trace elements with multiple associated health benefits.

## Figures and Tables

**Table 1:** Plasma carotenoid concentrations of volunteers at the time of recruitment with additional data from literature. Values represent means ± SD.

Study	Lutein (µg·L^−1^)	β-Carotene (µg·L^−1^)	Zeaxanthin (µg·L^−1^)
This study			
Day −14	107.2 ± 51.2 (range: 61.9–205.8)	199.0 ± 152.0 (range: 55.3–491.6)	17.3 ± 6.4 (range: 11.5–30.2)
Day −9	93.2 ± 44.9 (range: 63.1–180.9)	127.5 ± 64.9 (range: 34.1–401.5)	15.5 ± 5.2 (range: 8.0–24.9)
Day 0	74.8 ± 24.2 (range: 56.9–119.2)	104.6 ± 72.3 (range: 27.8–232.8)	13.5 ± 4.6 (range: 7.2–22.1)
Riso et al., 2002 [27]	386.7 ± 39.8	590.6 ± 64.4	39.8 ± 5.7
Stuetz et al., 2016 [18]	157.0	289.9	25.6

**Table 2:** Values (mean and range) obtained for AUC, C_max_ and t_max_ for each carotenoid of the 11 participants.

Carotenoid	Content in 6 g of Chlorella Vulgaris (mg)	AUC ^1^ (µg·h·L^−1^)	*p*-Value	C_max_^1^ (µg·L^−1^)	*p*-Value	t_max_^2^ (h)
Lutein	7.08	1001.6|141.5 * (235.3–4263.8)|(33.3–602.2) *	*p* < 0.01	20.4|2.9 * (4.8–86.4)|(0.7–12.2)*	*p* < 0.01	19.0 ± 15.7
β- Carotene	1.88	1302.4|692.8 * (338.3–5015.5)|(179.9–2667.8) *	*p* < 0.01	34.9|18.6 * (6.9–178.1)|(3.7–94.7)*	*p* < 0.01	34.9 ± 26.4
Zeaxanthin	1.47	122.2|83.1 * (25.5–585.8)|(17.3–56.5) *	*p* < 0.01	3.4|2.3 * (0.8–14.5)|(0.5–9.9)*	*p* < 0.01	22.5 ± 17.6

^1^ Values are presented as geometric mean (CI GEO 90% lower − CI GEO 90% upper); ^2^ values are presented as mean ± SD. * Values are standardized per mg of each carotenoid.

## Data Availability

All relevant data are within the manuscript files.

## Supplementary Materials

The following are available online at https://www.mdpi.com/article/10.3390/antiox10081164/s1, Supplementary Materials and Methods: Inclusion and exclusion criteria, Figure S1: Relative plasma concentrations of lutein during 3 days after intake of marine *Chlorella vulgaris* for the 11 volunteers, Figure S2: Relative plasma concentrations of β-carotene during 3 days after intake of marine *Chlorella vulgaris* for the 11 volunteers, Figure S3: Relative plasma concentrations of zeaxanthin during 3 days after intake of marine *Chlorella vulgaris* for the 11 volunteers, Figure S4: Variation of the urine ratios of the concentration of I/Creatinine and Se/Creatinine after 72 h of marine *Chlorella vulgaris* intake, Table S1: Nutritional and phytochemical composition of dried marine *Chlorella vulgaris*, Table S2: Prohibited and allowed foods during the study, Table S3: AUC values determined for the ratios of the concentrations of I/creatinine and Se/creatinine in urine between 0 and 72 h after marine *Chlorella vulgaris* intake.
