# Supplementary material for: A Single Dose of Marine Chlorella vulgaris Increases Plasma Concentrations of Lutein, β-Carotene and Zeaxanthin in Healthy Male Volunteers
Source: Antioxidants (Basel). 2021 Jul 22;10(8):1164. doi: 10.3390/antiox10081164 (PMC8388909; doi:10.3390/antiox10081164)
Supplement: Supplementary file 1 [file antioxidants-10-01164-s001.zip › antioxidants-1264973-supplementary.pdf]

Supplementary Material

A Single Dose of Marine *Chlorella vulgaris* Increases Plasma Concentrations of Lutein, β-Carotene and Zeaxanthin in Healthy Male Volunteers

Ana Teresa Serra, Sandra D. Silva, Luís Pleno de Gouveia, Agostinho M. R. C. Alexandre, Carolina V. Pereira, Ana Barbara Pereira, Ana Carvalho Partidário, Nuno Elvas Silva, Torsten Bohn, Vanessa S. S. Gonçalves, Gonçalo Real, Pedro Escudero, Naiara Fernández, Ana A. Matias, Maria Rosário Bronze

1. Supplementary Material and Methods

1.1. Inclusion and exclusion criteria

1.1.1. Inclusion criteria

1. Male subjects aged between 20 and 50 years.
2. There was no restriction related to body mass index (BMI).
3. Normal clinical record and physical examination.
4. Laboratory tests (hematology and biochemistry) within the range of normal values.
5. Not having participated in another clinical trial during the 3 months preceding the beginning of the present study.
6. Not having donated blood during the 4 weeks preceding the beginning of the study.
7. Free acceptance to participate in the study, with written informed consent form (ICF) of the volunteer.
8. Compliance with diet.

1.1.2. Exclusion criteria

Subjects who presented at least one of the following criteria were not eligible to participate in this study:
1. Regular microalgae consumers.
2. Allergy or intolerance to the test product.
3. Acute illness during the study.
4. Background or clinical evidence of chronic diseases such as anemia, cancer, renal insufficiency, history of chronic disease of the gastrointestinal tract, thyroid diseases, diabetes or others.
5. Daily consumption of alcohol >40g (>1L of beer or >4 glasses of wine per day).
6. Background of drug dependence within 3 months prior to the screening visit.
7. Taking or having taken antibiotics in the 3 months before recruitment.
8. Positive serology for hepatitis B, C or HIV.
9. Electrocardiogram abnormalities (clinically significant) or vital sign abnormalities (systolic blood pressure lower than 90 or over 180 mmHg, diastolic blood pressure lower than 50 or over 100 mmHg, or heart rate less than 50 or over 100 bpm) at screening.
10. Having undergone major surgery during the previous 6 months.
11. Tobacco consumption >10 cigarettes per day.

12. Being a consultant or employee of a company with direct interests in the supplement production or marketing.
13. Being a relative of principal investigator or employee of the investigational site (Cintramédica II – Serviços de saúde Lda) or employee iBET.
14. Students or others with any dependency relationship with the research team.
15. Nausea, vomiting or diarrhea.
16. Fail to adhere to the proposed diet.

2. Supplementary Tables

Table S1. Nutritional and phytochemical composition of dried marine *Chlorella vulgaris*.

Nutrition Facts/Composition	Per 100 g	Per dose (6 g)
Calories (KJ/Kcal)	1149/276	68.9/16.5
Lipids (g)	8.88	0.53
Saturated fatty acids (g)	1.4	0.08
Total Carbohydrate (g)	5	0.3
Sugar (g)	1.64	0.10
Fiber (g)	22.2	1.3
Proteins (g)	32.8	1.9
Salt (g)	14.8	0.8
**Carotenoids**		
Lutein	118 mg	7.08 mg
β-Carotene	31.4 mg	1.88 mg
Zeaxanthin	24.1 mg	1.47 mg
**PUFAs**		
α-Linolenic acid	1.45 g	87 mg
Linoleic acid	1.08 g	64.8 mg
EPA	55 mg	3.3 mg
DHA	48 mg	2.88 mg
**Mineral Salts**		
Se	5.1 μg	0.3 μg
I	1.32 mg	79.2 μg

Table S2. Prohibited and allowed foods during the study.

	Prohibited foods throughout the study	Prohibited foods during the 3 days prior to the product intake	Allowed foods during experimental phase
**Vegetables**	Carrot<br>Tomato<br>Broccoli<br>Spinach<br>Brussel sprouts<br>Cress<br>White and green cabbage<br>Lettuce<br>Avocado<br>Pepper<br>Peas<br>Asparagus	Carrot<br>Tomato<br>Broccoli<br>Spinach<br>Brussel sprouts<br>Cress<br>White and green cabbage<br>Lettuce<br>Avocado<br>Pepper<br>Peas<br>Asparagus	Potato<br>Legumes (grain, lentils, beans)<br>Mushrooms
**Fruits**	Apricot<br>Citrus<br>Mango<br>Melon<br>Watermelon<br>Papaya	Apricot<br>Citrus<br>Mango<br>Melon<br>Watermelon<br>Papaya	Banana<br>Strawberry<br>Cherry<br>Apple
**Fish**		Salmon<br>Tuna fish<br>Sardines<br>Hake<br>Mackerel	-
**sSellfish**	-	Shrimp<br>Mussel<br>Clams<br>Oysters	-
**Meat**	-	-	Chicken<br>Turkey<br>Pig<br>Cow<br>Rabbit
**Others**	Eggs	Eggs<br>Seeds<br>Dry fruits<br>Oat<br>Soy<br>Oil	Rice<br>Pasta<br>Dairy products

Table S3. AUC values determined for ratio I/creatinine and Se/creatinine in urine between 0 h and 72 h after marine *Chlorella vulgaris* intake.

	Se	I
**AUC (µg.h/g)**	55.9 ± 521.7	526.8 ± 781.8
**p value (AUC≤0)***	0.730	0.049

*Student's paired t-test

3. Supplementary Figures

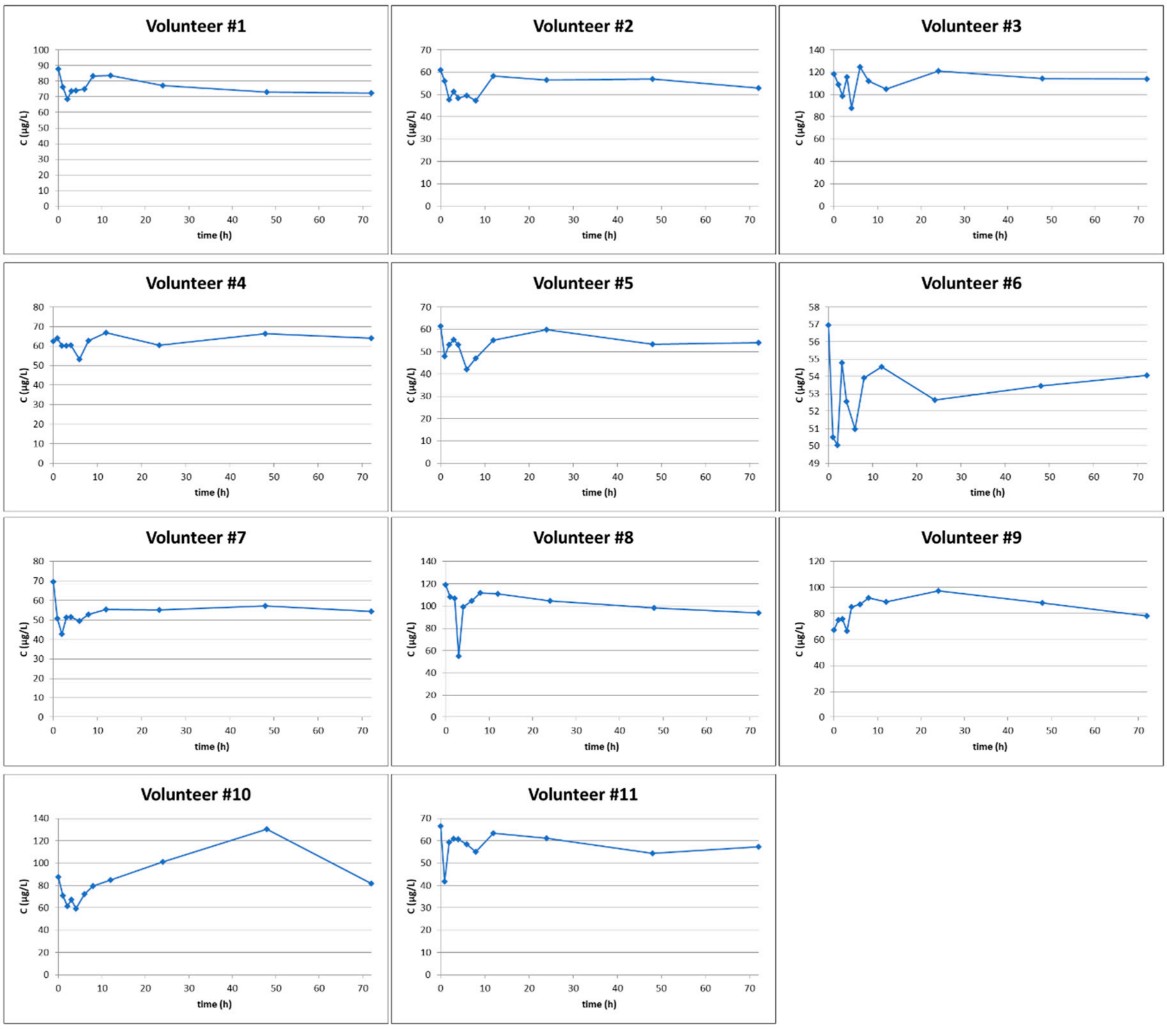

Figure S1. Plasma concentrations of lutein during 3 days after intake of marine *Chlorella vulgaris* for the 11 volunteers.

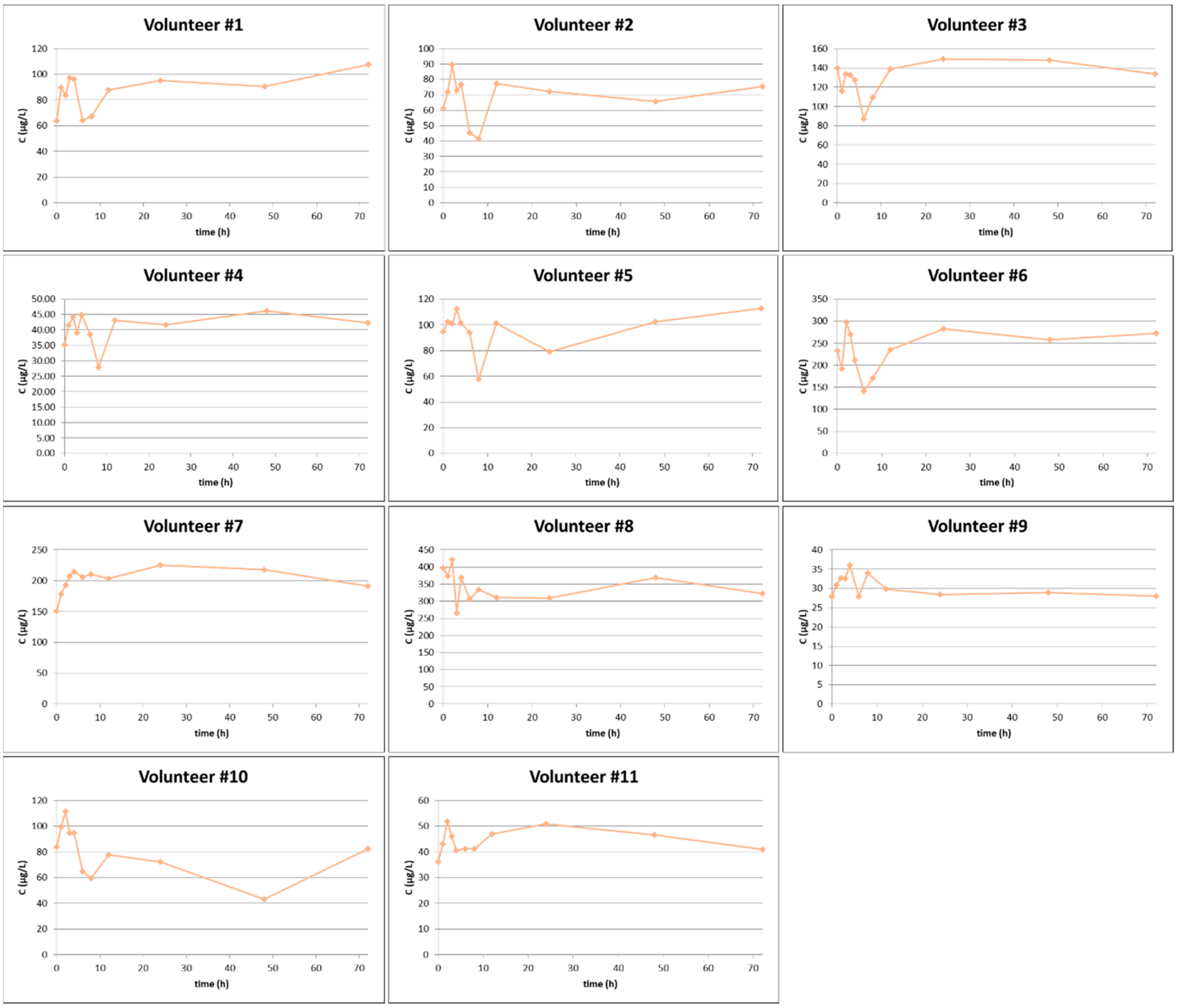

Figure S2. Plasma concentrations of β-carotene during 3 days after intake of marine *Chlorella vulgaris* for the 11 volunteers.

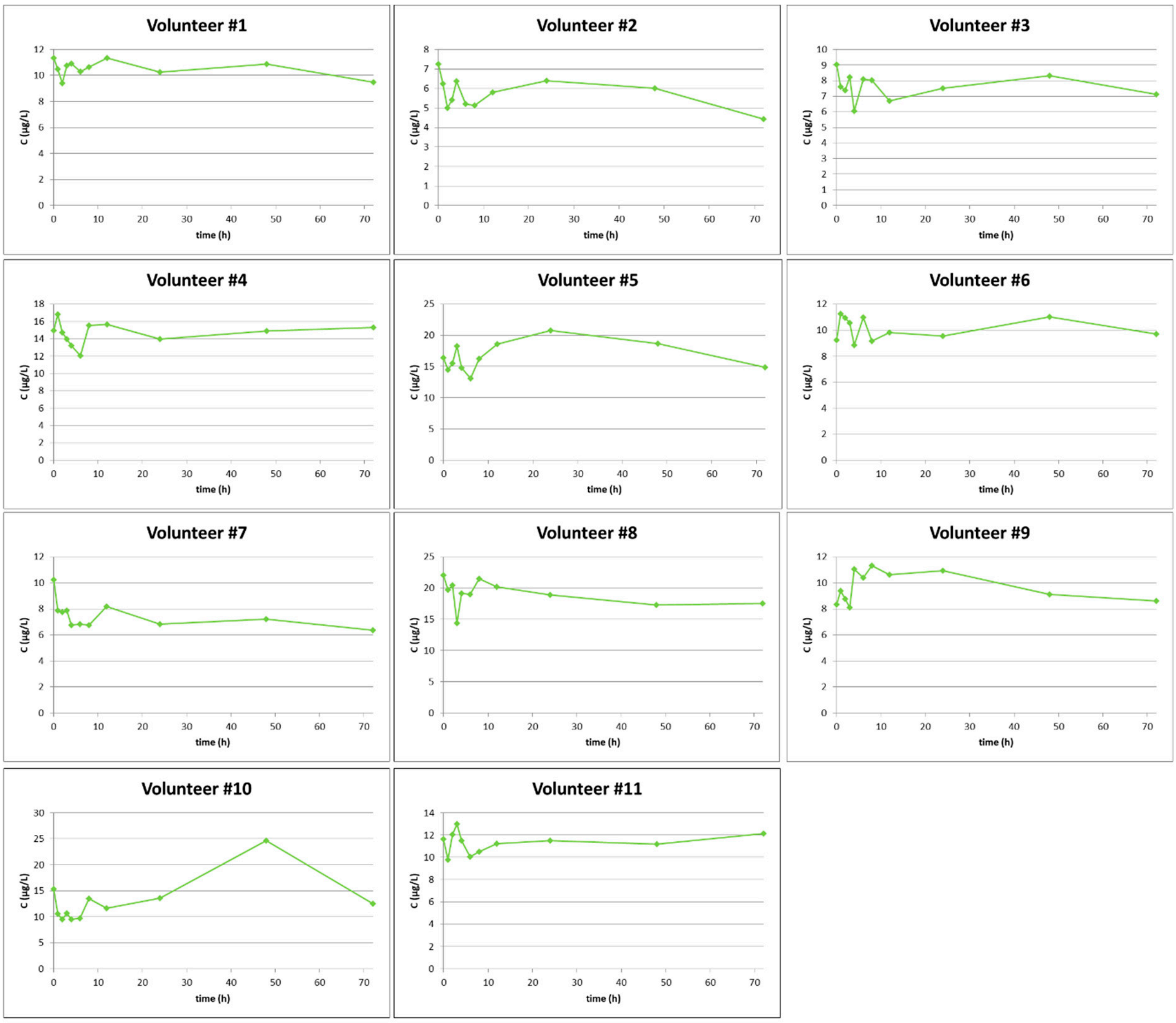

Figure S3. Plasma concentrations of zeaxanthin during 3 days after intake of marine *Chlorella vulgaris* for the 11 volunteers.

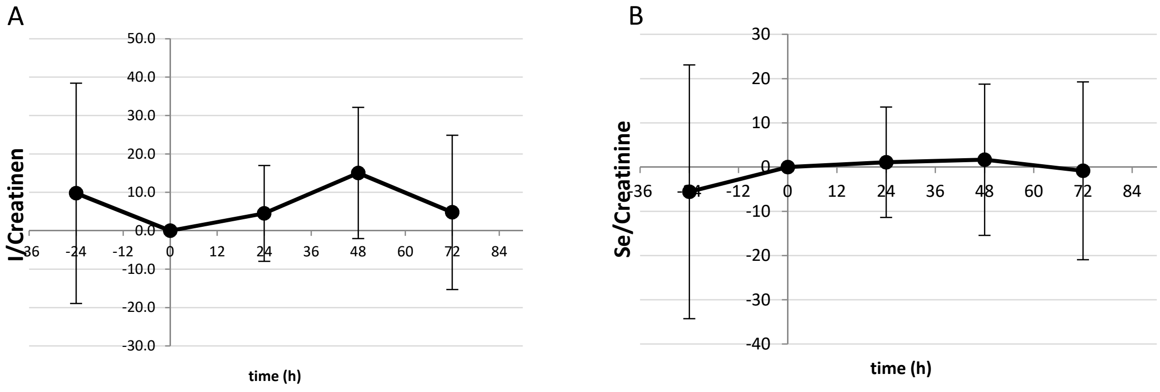

Figure S4. Variation of the urine ratios of the concentrations of I/Creatinine (A) and Se/Creatinine (B) (mass base) after 72 h of marine *Chlorella vulgaris* intake. (Note: the baseline value of -9 days was presented as 24 h to improve the visual interpretation of the profile between 0 and 72 h.) Error bars present the SD of 11 subjects.
